# Securing the future of Europe's ageing population by 2050

**DOI:** 10.1016/j.lanepe.2023.100807

**Published:** 2023-12-01

**Authors:** The Lancet Regional Health – Europe

The global population is experiencing a rapid increase in the older demographic, both in terms of numbers and proportion of the total population. Concurrently, the younger, working-age demographic is in decline. Currently, in the European Union (EU), more than a fifth of the population is aged 65 years or older, with a ratio of three working-age individuals to each older person. However, projections suggest that older individuals will account for nearly a third of the population by 2050, with less than two working-age individuals for every older person.

The ageing population in Europe by 2050 will have profound implications across various sectors, including health care, social welfare, the labour market, and the economy. This demographic shift will substantially increase the burden of chronic diseases and age-related conditions, placing substantial financial strain on health-care systems. Social welfare systems, including pensions and long-term care services, will face increasing pressure. A shrinking workforce will lead to labour shortages, potentially necessitating the extension of retirement age or encouraging the participation of older workers. An older population could lead to reduced economic growth, productivity, and consumption, potentially slowing economic development.

These changes will undoubtedly impact the wellbeing of society as a whole and must be addressed proactively. It is imperative to explore effective approaches to secure the future of Europe's ageing population. A well-planned, holistic approach, addressing short-term, medium-term, and long-term goals, is needed to ensure the wellbeing and prosperity of future generations.

In the short term, the priority should be to preserve the health and productivity of ageing individuals. As people grow older, they face emerging and chronic health issues, frailty, and a decline in functionality. Non-communicable diseases account for 74% of all deaths. When individuals have multiple non-communicable diseases, they become more susceptible to co-morbidities and other diseases, resulting in more severe outcomes. To address this, it is essential to focus on non-communicable diseases clinics, which specialise in risk control, symptom management, and rehabilitation. Moreover, age-associated declines in physical functions, leading to common issues like hearing loss and cataracts, restrict the productivity and independence of older individuals. Addressing these challenges requires easily accessible and affordable treatments.

In the medium term, the objective should be to enhance senior-friendly living. Vienna, recently recognised as the most livable city, is actively pursuing this goal to enable older people to live a self-determined life and strengthen senior citizens' participation in the community. Vienna's commitment to creating an age-friendly environment is evident through measures such as a robust network of care services, diverse recreational activities, affordable housing, support and social benefits, accessible information, and an extensive public transport network. This initiative in Vienna serves as an inspiring model for other countries.

The long-term goal should be to boost the fertility rate in response to the decline in birth rates observed in Europe. Fertility rates across European member states have converged to a critically low level, averaging just 1.53 livebirths per woman in 2021, far below the critical 2.1 livebirths per woman that is needed to maintain a population in equilibrium. Strategies including childcare support, paternal leave incentives, flexible work schedules, universal health care have shown gradual positive effects on fertility rates, as exemplified by France, which has the highest fertility rate in Europe (1.84 livebirths per woman in 2021). While immediate population growth might not occur, a steadfast commitment to these family-friendly policies is essential to encourage people to have children.

In addition to the short-term, medium-term, and long-term strategies, migrant health in Europe must be addressed expeditiously to ensure the wellbeing and vitality of the continent. Many countries will increasingly rely on immigration to address their demographic imbalances. At present, the WHO European region accommodates the largest proportion of international migrant populations: approximately one in eight people are refugees or migrants. This number is expected to rise by 2050. This emphasizes the urgent need to invest in and improve migrant health. A comprehensive approach that considers health along the entire migration route is essential, since the health of migrants will significantly contribute to the overall wellbeing of future European populations.

It is noteworthy that as early as 2024, the number of individuals aged over 65 years is projected to surpass those under the age of 15 in the WHO European Region. Consequently, Europe must proactively prepare for a future dominated by an aging population, challenging traditional stereotypes of frailty and dependence. These short-term, medium-term, and long-term goals, combined with a focus on migrant health, should be embraced by all stakeholders, including governments, civil society, and policymakers at all levels. Their collective efforts are essential to safeguard the health, productivity and wellbeing of the aging population by 2050.

